# Circr, a Computational Tool to Identify miRNA:circRNA Associations

**DOI:** 10.3389/fbinf.2022.852834

**Published:** 2022-03-11

**Authors:** Martina Dori, Jimmy Caroli, Mattia Forcato

**Affiliations:** ^1^ Department of Life Sciences, University of Modena and Reggio Emilia, Modena **,** Italy; ^2^ Department of Drug Design and Pharmacology, University of Copenhagen, Copenhagen, Denmark

**Keywords:** circRNA, miRNA, interactions prediction, miRNA binding, prediction tools

## Abstract

Circular RNAs (circRNAs) are known to act as important regulators of the microRNA (miRNA) activity. Yet, computational resources to identify miRNA:circRNA interactions are mostly limited to already annotated circRNAs or affected by high rates of false positive predictions. To overcome these limitations, we developed Circr, a computational tool for the prediction of associations between circRNAs and miRNAs. Circr combines three publicly available algorithms for *de novo* prediction of miRNA binding sites on target sequences (miRanda, RNAhybrid, and TargetScan) and annotates each identified miRNA:target pairs with experimentally validated miRNA:RNA interactions and binding sites for Argonaute proteins derived from either ChIPseq or CLIPseq data. The combination of multiple tools for the identification of a single miRNA recognition site with experimental data allows to efficiently prioritize candidate miRNA:circRNA interactions for functional studies in different organisms. Circr can use its internal annotation database or custom annotation tables to enhance the identification of novel and not previously annotated miRNA:circRNA sites in virtually any species. Circr is written in Python 3.6 and is released under the GNU GPL3.0 License at https://github.com/bicciatolab/Circr.

## Introduction

Circular RNAs (circRNAs) (Salzman et al., 2012; Hansen et al., 2013a; Jeck et al., 2013; Memczak et al., 2013) are covalently closed RNA molecules characterized by a non-linear 3′-5′ junction resulting from an unusual splicing event denominated backsplice junction (Ashwal-Fluss et al., 2014; Lasda and Parker, 2014; Conn et al., 2015; Ivanov et al., 2015; Starke et al., 2015; Wang and Wang, 2015; Zhang et al., 2016). This particular splicing event causes circRNAs to lack both 3′ poly(A) tail and 5′ capping, conferring resistance to exonuclease activity [e.g., RNaseR (Suzuki et al., 2006; Vincent and Deutscher, 2006)] and therefore determining a general longer half-life as compared to linear RNAs (Jeck et al., 2013). Despite the great interest towards this class of non-coding RNA especially for their potential as disease biomarkers (Li et al., 2015; Memczak et al., 2015; Lyu and Huang, 2017; Jahani et al., 2020; Wang et al., 2021), only few of them have been functionally characterized to date [a few examples are reported in (Memczak et al., 2013; Ashwal-Fluss et al., 2014; Barrett and Salzman, 2016; Suenkel et al., 2020)]. Several biological functions have been proposed for circRNAs, ranging from the regulation of pluripotency to early lineage differentiation (Yu et al., 2017; Izuogu et al., 2018) to the control of unique functions in specialized cells (Berta et al., 1990; Capel et al., 1993). However, in terms of research interest, the most prominent function attributed to circRNAs is their action as miRNA sponge to regulate target gene expression by inhibiting miRNA activity through competition with the RISC complex, a function commonly defined as miRNA binding (Hansen et al., 2013a; Hansen et al., 2013b; Memczak et al., 2013; Huang et al., 2017; Panda, 2018; Verduci et al., 2019).

The computational resources that have been recently developed to investigate circRNA function and expression substantially consist of databases collecting all available information relative to already known circRNAs in different species [e.g., circBase (Glažar et al., 2014)]. Although these databases have been expanded to account for circRNA expression patterns, functional predictions (e.g., miRNAs binding sites) and disease associations (Yang et al., 2011; Ghosal et al., 2013; Li et al., 2014; Dudekula et al., 2016; Liu et al., 2016), still they are limited to previously identified circRNAs in human and mouse species only. Moreover, most of the available tools to predict miRNA:circRNA interactions fall short when dealing with previously uncharacterized circRNAs. For instance, some resources, requiring a Gene ID as input and not permitting sequence browsing, can predict miRNA targets only for annotated genes and are therefore informative only if circRNAs include a 3′ UTR. On the other hand, tools that allow the analysis of sequences are restricted to a fixed nucleotide sequence length limiting the number of sequences that can be investigated at once (Kertesz et al., 2007; Betel et al., 2008; Rennie et al., 2014; Agarwal et al., 2015; Rennie et al., 2016; Karagkouni et al., 2018). In an attempt to bypass these limitations, some prediction tools provide offline stand-alone versions of their algorithms (Enright et al., 2003; Rehmsmeier et al., 2004; Thadani and Tammi, 2006; Bandyopadhyay and Mitra, 2009; Hsu et al., 2011; Coronnello and Benos, 2013; Agarwal et al., 2015). This solution, while allowing a full customization of the analysis, comes with the drawback of a high rate of false positives as these approaches easily return thousands of putative miRNA:RNA interactions. A common solution to reduce these lists is to perform a systematic validation of the targets (e.g., with RIP assays) or the integration with known interactions. In particular, since the binding of the RISC complex is mediated by the interaction of the miRNA with members of the Argonaute protein family (Hammond et al., 2000; Hutvágner and Zamore, 2002), it is fundamental, for a predicted miRNA:circRNA site to be functional, that also Ago proteins are binding in the same positions. Currently, the development of various CLIP-Seq (Cross-linking and Immunoprecipitation followed by sequencing) protocols provides an extremely valuable source of high-throughput data for Ago binding sites (Chi et al., 2009; Hafner et al., 2010; Jungkamp et al., 2011; Leung et al., 2011; Helwak et al., 2013; Clark et al., 2014; Grosswendt et al., 2014). In addition, the availability of already validated miRNA:RNA sites obtained through RIP assays (or other experimental approaches) provides a further layer of valuable information for filtering candidate miRNA:circRNA pairs. Following the approach proposed in (Dori and Bicciato, 2019), we developed Circr, a tool that integrates miRNA:target predictions from three commonly used algorithms, i.e., miRanda (Agarwal et al., 2015), TargetScan (Enright et al., 2003), and RNAhybrid (Rehmsmeier et al., 2004) with validated miRNA:RNA interactions and AGO peaks data. The combination of multiple tools for the identification of a single miRNA recognition site together with experimental data allows to efficiently reduce the pool of candidate miRNA:circRNA interactions for functional studies.

## Methods

### Implementation

Circr is written in Python3 and is compatible with Linux, Mac OS, and the MS Windows subsystem for Linux. The tools is freely available from Github (https://github.com/bicciatolab/Circr) and depends on several modules (pandas, pybedtools, collections, multiprocessing, functools, itertools, operator) that can be installed *via* pip. Circr runs three different software for miRNA target prediction, namely miRanda (Agarwal et al., 2015), TargetScan (Enright et al., 2003), and RNAhybrid (Rehmsmeier et al., 2004).

miRanda (http://www.microrna.org/microrna/getDownloads.do) and RNAhybrid (https://bibiserv.cebitec.uni-bielefeld.de/rnahybrid/) must be downloaded and installed by the user from their respective official source; TargetScan is included in the Circr package as a standalone perl script. Circr supports single core and multithreading calculations. We suggest running Circr on a machine with at least 8 GB of RAM.

Circr comprises an internal annotation database that can be downloaded from the following folder https://drive.google.com/drive/folders/1zJVyzEFAMtvZTTueWRocxXs63jUxsl-U?usp=sharing (refer to the README file for details on the database content). This database includes the following information for *H. sapiens*, *M. musculus*, *D. melanogaster*, and *C. elegans*: 1) the genome sequence in FASTA format; 2) the ENSEMBL (Howe et al., 2021) gene annotation in GTF format and the rRNA coordinates; 3) the miRNA sequences from the latest version (v22) of miRBase (Kozomara et al., 2019); 4) the annotation files in BED format with the experimentally validated miRNA:RNA pairs (Grosswendt et al., 2014; Li et al., 2014; Luna et al., 2015; Moore et al., 2015; Luna et al., 2017; Karagkouni et al., 2018; Kobayashi et al., 2019); and 5) the Argonaute peaks coordinates (Moshkovich et al., 2011; Consortium, 2012; Huang et al., 2013; Balakrishnan et al., 2014; Boudreau et al., 2014; Erhard et al., 2014; Pillai et al., 2014; Rybak-Wolf et al., 2014; Moore et al., 2015; Gillen et al., 2016; Rayon-Estrada et al., 2017; Benway and Iacomini, 2018; Nowakowski et al., 2018; Sarshad et al., 2018; Xiao et al., 2019; Xu et al., 2019; Fernandes et al., 2021); see Supplementary Table S1 for the complete list of organism and genome versions provided. miRNA:RNA interactions and AGO peaks were retrieved from TarBase (Karagkouni et al., 2018) and ENCORI (Li et al., 2014) databases or they were available as supplementary data files in the Gene Expression Omnibus (https://www.ncbi.nlm.nih.gov/geo/) series associated to the referenced publications. Then, we merged the files according to organism and genome assembly and finally used the UCSC Genome Browser tool LiftOver (Kent et al., 2002) to obtain the coordinates for the different genome versions (necessary files were downloaded from the Genome Browser Download Page http://hgdownload.soe.ucsc.edu/downloads.html).

### Pipeline Overview

The core of Circr consists of three main steps: 1) sequence extraction from circRNA genomic coordinates; 2) prediction of miRNA:circRNA interactions with third party software; and 3) comparison of the predicted sites with a database of validated interactions and AGO peaks.

### circRNAs Sequence Extraction

**FIGURE 1:**
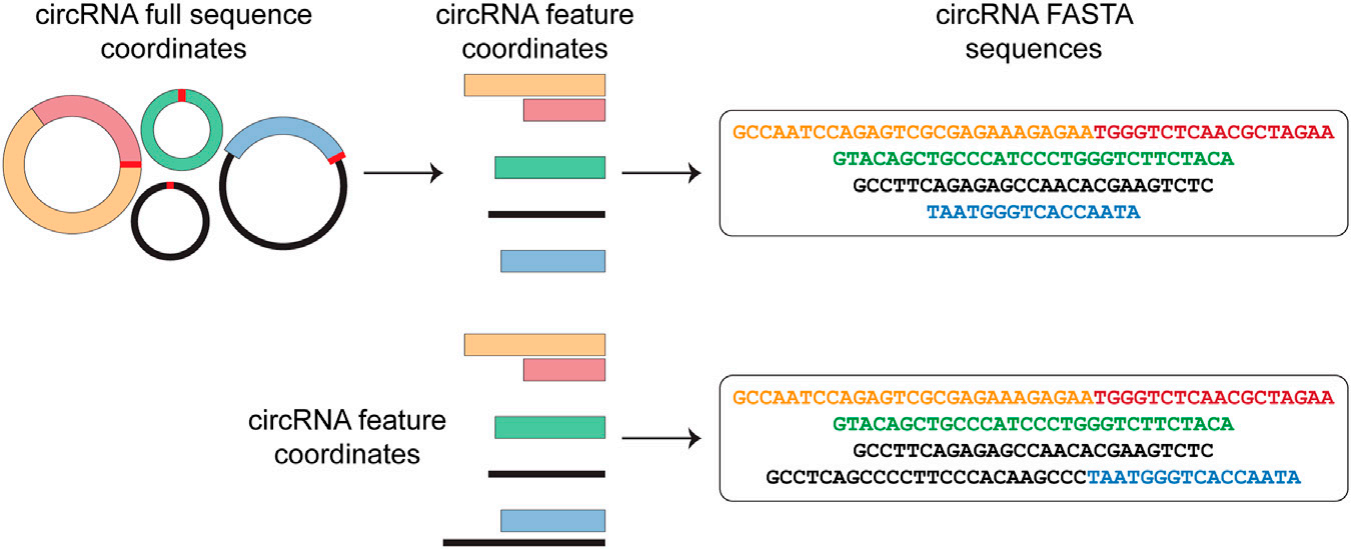
circRNAs sequence extraction. As a first step, Circr takes a set of coordinates in BED format as input. By default, it assumes that the transcripts overlapping genes on the same strand include only exons, therefore it retrieves the coordinates of the included features and uses them to construct the FASTA sequence of each circRNA. If the input file includes the exact composition of the circRNA (or if users want to avoid the automatic exon-only selection), Circr skips the initial exon/intron splitting and uses the supplied coordinates to retrieve the FASTA sequence.

Circr requires in input a BED file containing the circRNAs genomic coordinates. Starting from this file, Circr generates a data frame of nucleotide sequences in FASTA format (Figure 1). By default, Circr assumes that the input coordinates are the coordinates of the circRNAs full sequences (i.e., containing both introns and exons) that need to be processed prior to sequence extraction. For this purpose, Circr compares circRNAs coordinates with gene annotation. If circRNAs overlap genes annotated on the same strand, they are split into intron/exon coordinates and only the coordinates of the latter are retained for sequence retrieval. Conversely, antisense and intergenic circRNAs are considered as a single exon. By providing the organism and genome build required for the analysis, the tool automatically retrieves the necessary files from its internal database. Circr also accepts custom reference files provided by the user to maximize the applicability of the method to different organisms or genome builds (for more details and examples on running Circr with custom files, see https://github.com/bicciatolab/Circr#running-circr-with-custom-annotation-files). As for linear mRNA, circRNAs that overlap genes can undergo alternative splicing, implying that they might not include all exons within the transcript start/end coordinates or might retain introns. Therefore, the assumption of an exon-only structure could lead to an erroneous analysis and loss of information. To avoid this pitfall, Circr can accept as input a set of coordinates of all the features (exon/intron) known to be included in each circRNA of interest (circRNA features). In this case, Circr skips the initial intron/exon splitting. This option can also be used to avoid exon/intron splitting when there is no prior knowledge of the actual circRNA sequence structure, thus considering each circRNA as a single exon. Once all the genomic coordinates of the provided circRNAs have been computed, Circr proceeds to extract their sequences in FASTA files.

### Prediction of miRNA:circRNA Interactions

**FIGURE 2:**
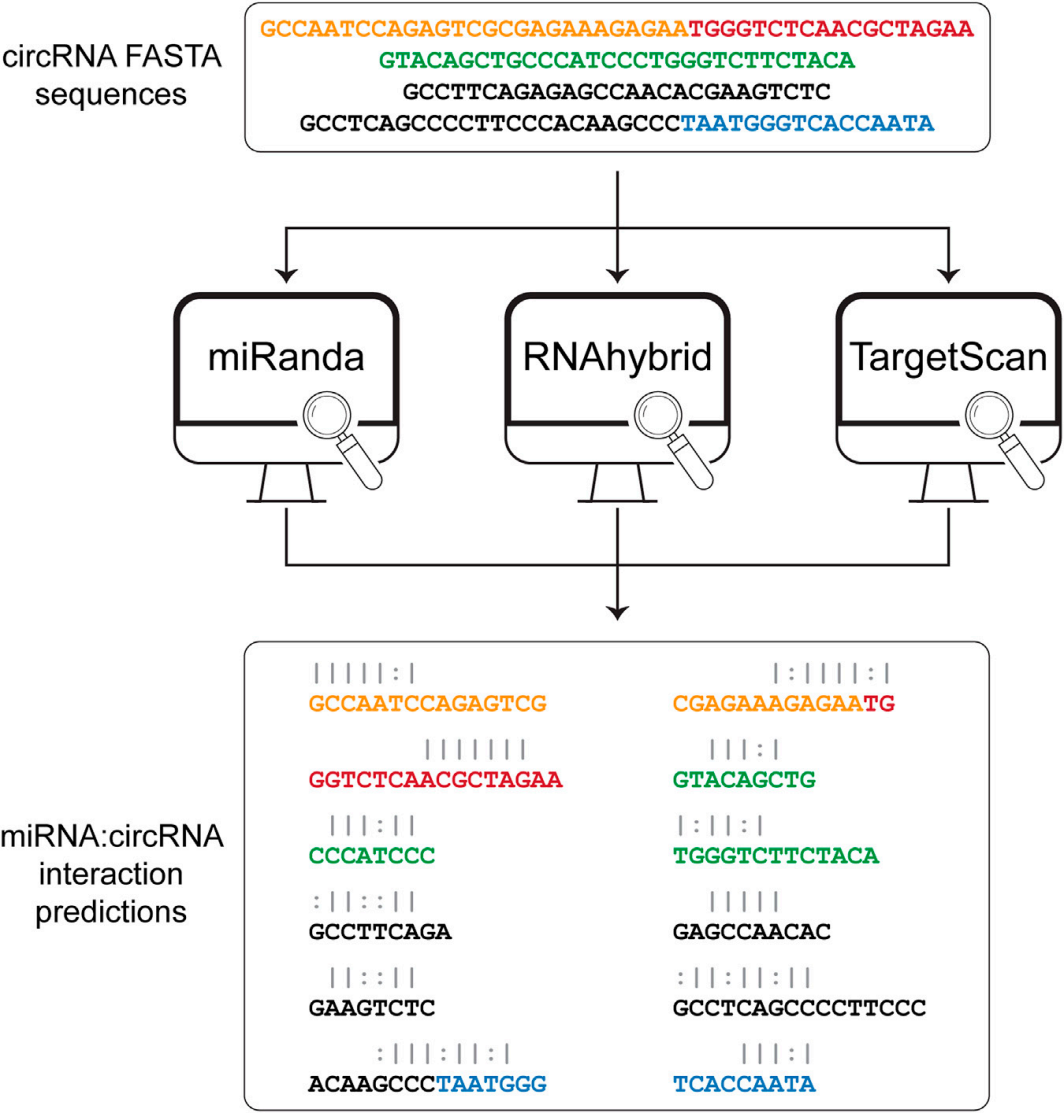
Prediction of miRNA:circRNA interactions. The list of FASTA sequences generated in the first step is then given as input to each of the 3 tools that are included in Circr, i.e., miRanda, RNAhybrid, and TargetScan. The result for each tool is then parsed and collected in a comprehensive result table where only unique miRNA:circRNA interacting sites are retained. White spaces indicate a mismatch, pipe represents a match while colon represent a G-U pair.

The sequences obtained from the sequence extraction step are used as input to three algorithms for the prediction of the miRNA binding sites (Figure 2). Among the various available tools for miRNA binding site prediction, we focused on the most commonly used, i.e., miRanda (Agarwal et al., 2015), RNAhybrid (Rehmsmeier et al., 2004), and TargetScan (Enright et al., 2003), as they all provide a standalone version that can be easily downloaded and installed on any Unix-based system. All three tools work primarily on sequence complementarity (seed match). Specifically, miRanda accounts for the free energy of the duplex together with seed sequence conservation and position and calculates a final score based on base match and gap penalties. RNAhybrid evaluates only the free energy from each possible match between the short (miRNA) and long (circRNA) sequences, retaining only the most stable duplexes. TargetScan searches for 8mer, 7mer, and 6mer on the target RNA that match the miRNA seed sequence. Circr runs each tool with default parameters and without applying any filter on the resulting output. To optimize the computation time, the three algorithms are run as parallel sub processes. Subsequently, the output files of each tool are first collected and then the predicted interactions are converted back to genomic coordinates and annotated using the miRNA:RNA seed category described in (Bartel, 2009) (e.g., 8mer or 7mer seed sequence). Finally, all processed outputs are collected into a table reporting also the number of tools that were able to identify that specific miRNA binding site.

### Comparison of Predicted Interaction Against Validated Databases

**FIGURE 3:**
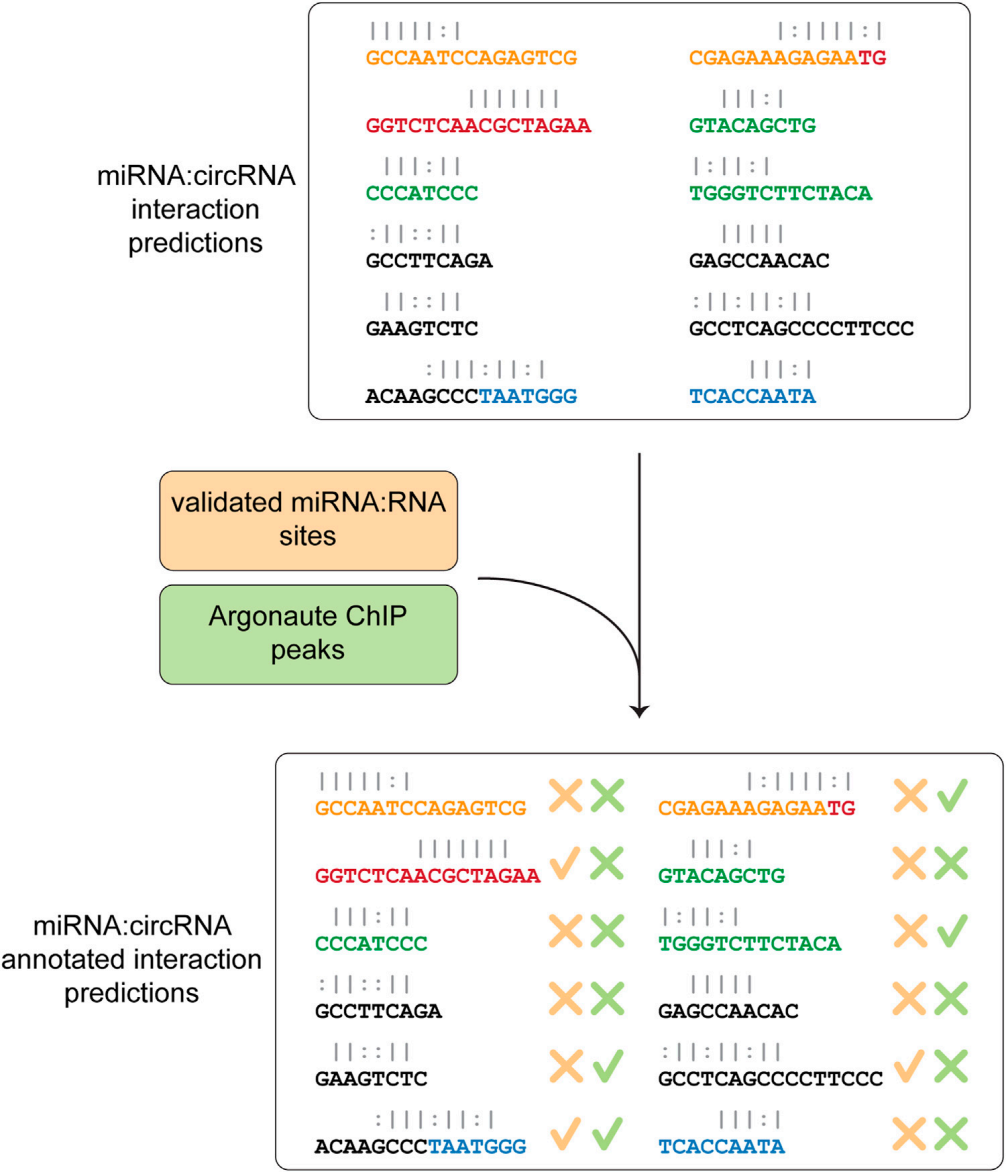
Comparison of predicted interaction against validated databases. The final step consists in the annotation of the predicted miRNA:circRNA interactions with experimentally validated miRNA-RNA sites and peaks of Argonaute binding. The final table is then saved as a CSV format file.

In the last step, Circr compares the predicted interactions against the collection of publicly available and experimentally validated miRNA:RNA pairs as well as AGO peaks coordinates contained in the internal database. This comparison is central to select potentially relevant miRNA:circRNA pairs, as it focuses on seed regions that were either validated (and therefore there is a known direct interaction among the two RNAs) or overlap an Argonaute binding site (meaning that the RISC complex is potentially recruited to that specific site). The final table reports whether there is an overlap between the coordinates of validated seed regions or AGO peaks and the predicted ones (Figure 3). Although Circr comprises an internal database of validated interactions and AGO peaks derived from publicly available data, the user can provide custom sets of interactions to focus the analysis on specific sequences of interest.

### Output Format

As output, Circr generates a table that allows the user to easily explore and filter out the seed sequences of interest. The output table consists of 12 columns containing the genomic coordinates of the miRNA binding site, the miRNA name, the investigated circRNA, and its strand, the inferred seed category, a unique interaction identifier, the number of software that have predicted the interaction, flags specifying whether the predicted interaction is experimentally validated or overlaps an Argonaute peak and, if the circRNA coordinates are reported in circBase (http://www.circbase.org/), the corresponding circBase ID (Table 1).

**TABLE 1:** Field description of the output file generated by Circr.

Field	Description	Example
Chrom	circRNA chromosome	chr1
Start	Start coordinate of the miRNA:circRNA interaction	16453189
End	End coordinate of the miRNA:circRNA interaction	16453211
miRNA Name	miRNA name as specified in miRBase (v22)	mmu-miR-3101-3p
Circ Name	circRNA ID	CiCo_mm9_circ_000139
Strand	circRNA strand	-
Seed Category	Categorization of miRNA seed according to Bartel, (2009)	7mer-m8
ID	Unique interaction ID. The letter in the middle specifies the tool that identified the interaction (M for miRanda, RH for RNAhybrid and TS forTargetScan)	INT_M_3224
Software Matched	Number of tools that identified the same interaction	3
Validated	Yes/No flag to indicate if the identified site has been previously validated	Yes
AGO	Yes/No flag to indicate if the site overlaps an Argonaute peak	No
circBase ID	If present in circBase, the relative ID is provided	mmu_circ_0000012

### Testing on Example Dataset

To assess Circr performance and running time, we analyzed 100 circRNAs identified in the developing mouse brain, selected from Supplementary Data S1 of (Dori et al., 2019). These analyses were performed on both a server running Ubuntu 18.04.4 and a computer running macOS 10.15, using 8 CPUs.

## Results

**FIGURE 4:**
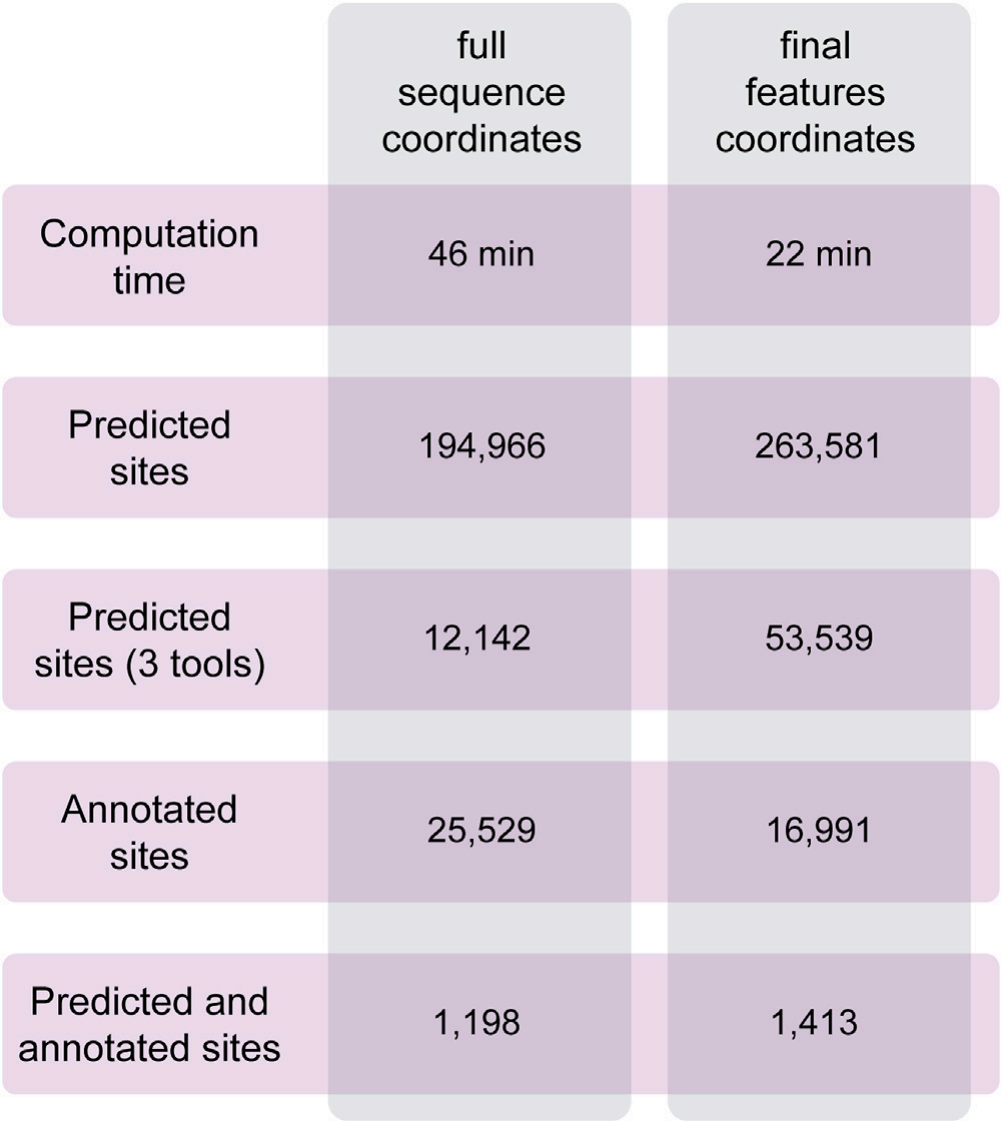
Summary of the results obtained with Circr on a server with Ubuntu 18.04. The analysis was performed on 100 circRNAs selected from the list of transcripts expressed in the lateral cortex of the mouse developing brain. The BED file including the coordinates of the chosen circRNAs was supplied both as the full sequence and as the set of final features.

To test the performances of Circr, we analyzed a set of circRNAs predicted to be expressed in the developing mouse brain (Dori et al., 2019). Data were obtained from RNAseR treated RNA of the three main cell population of the lateral cortex (proliferating and differentiating progenitors and newborn neurons). From this cohort of sequences, we selected 100 circRNAs of different lengths overlapping genes in the sense and antisense strand, and intergenic ones to cover all possible genomic features of circRNAs. As described in the Methods section, it is possible to provide in input the coordinates of the full sequence of the circRNA and let Circr calculate the exon coordinates or a final set of coordinates with all the features included in each circRNA. To test Circr, we used the 100 mouse sequences and analyzed them using both types of input (i.e., full sequence and final set of coordinates), with the default set of support files relative to the mm9 mouse genome. When providing the genomic coordinates of circRNAs full sequences, Circr completed the analysis in 46 min and retrieved more than 190,000 miRNA:circRNA binding sites (Figure 4). Conversely, providing the input file as a set of final exon/intron coordinates, Circr took 227 min for the prediction of 263,581 sites (Figure 4). Starting from Circr output, we can either focus on interactions identified by all 3 algorithms, resulting in 12,142 hits for the full sequence analysis and 53,539 for the feature coordinates one, or we can select only those overlapping either validated seed regions or an AGO peak, obtaining a list of 25,529 sites (32 if we account for the presence of both an AGO peak and a validated interaction) from the full sequence analysis and 16,991 (20 with both AGO/validated interaction) for the feature coordinates set up. By combining all criteria (3 tools and either an AGO peak or a validated interaction), the list goes down to 1,198 and 1,413 interaction sites for the full sequence and feature coordinates respectively, providing the user with a more manageable number of putative interacting miRNA:circRNA pairs for further validation. The analyses were carried out on an Ubuntu server using 8 threads. The same analyses performed on a personal computer with MacOS 10.15 and 8 threads required 132 min for the full sequence and 384 min for the final set input.

## Discussion

Circular RNAs have emerged as a significant player in both physiological and pathological conditions and have become an invaluable source of information as prognostic markers in cancer and several other diseases. However, despite a large number of circRNA databases is currently available, their limitation to already known and annotated sequences in specific organisms hampers the identification of novel circRNAs and the investigation of their potential role as miRNA regulators. As an example, CircNet 2.0 (Liu et al., 2016) focuses on circRNAs found in human cancers whereas ENCORI (Li et al., 2014) collects circRNAs only from human and mouse. Other limitations reside in the nature of miRNA:circRNA interactions reported and their annotation: CircNet 2.0 reports only interaction predicted by at least one tool among PITA, miRanda and TargetScan, but provides only the miRNA ID without specifying the number and genomic location of binding sites. The reported interactions are all supported by miRTarBase (Huang et al., 2020) but these validations involve the miRNA and the gene hosting the circRNA, irrespectively of the site of the interaction. ENCORI reports interactions predicted by just a single tool (miRanda) and only if supported by Ago CLIP-seq Data. From a usability point of view, ENCORI is miRNA-centered and does not allow searching by circRNA ID or coordinates. To cope with these needs, we developed Circr, a tool that combines the output of different miRNA binding site prediction algorithms with validated miRNA:RNA interactions (through direct approaches as luciferase assays or RNA-IP) and Argonaute binding site data. The output of Circr returns a comprehensive view of the circRNAs interactome, it has been structured to be easily browsed and investigated, thus helping researchers to narrow down the list of potential targets for further validation and functional characterization. We applied Circr to the analysis of a set of circRNAs predicted to be expressed in the developing mouse brain and, among the interactions supported by a validated seed region or an AGO peak, we focused our attention to those involving miRNAs known to be expressed in the same tissue (as, for instance, miR-9, miR-124, miR92a, miR92b). As a confirmation, we found a binding site identified by all 3 algorithms for miR-9 on CiCo_mm9_circ_000349, a circRNA already annotated in circBase (mmu_circ_0000044) and identified in mouse brain by (Memczak et al., 2013; Rybak-Wolf et al., 2015). In addition, we also found a binding site, predicted by 2 tools, for miR-92a/miR-92b on CiCo_mm9_circ_002328 (mmu_circ_0006166). To compare Circr performance on a circRNA reported in existing databases, we interrogated CircNet 2.0 and ENCORI for miRNA bindings of hsa_circ_0001946 (CDR1as), a well characterized human circRNA. Circr results reproduced the findings of the two databases and was able to identify two validated interactions with mir-7 and mir-671 (Hansen et al., 2011; Memczak et al., 2013).

One of the major features of Circr resides in its flexibility, as users can virtually investigate any given sequence in any given organism, as long as all the necessary files are available and provided. A possible limitation of our method could be represented by the fact that, in its current implementation, Circr adopts exclusively prediction tools based on seed matching, a feature that might affect the completeness of the information provided. However, we foresee that the growing interests toward this class of RNA will lead to the development of more refined and complete databases coupled with dedicated computational approaches that could be easily incorporated in Circr. In conclusion, although exploiting a previously described approach [e.g., (Chen et al., 2021)], to date Circr represents the first standalone tool that combines multiple prediction algorithms together with experimental data and we believe that it can provide a relevant contribution to the discovery of new and potentially functionally relevant miRNA:circRNA interactions.

## Data Availability

Publicly available datasets were analyzed in this study. This data can be found here: https://github.com/bicciatolab/Circr.
