# Supplementary material for: Circr, a Computational Tool to Identify miRNA:circRNA Associations
Source: Front Bioinform. 2022 Mar 11;2:852834. doi: 10.3389/fbinf.2022.852834 (PMC9580875; doi:10.3389/fbinf.2022.852834)
Supplement: Supplementary file 1 [file Table1.DOCX]

**Supplementary Table 1.** List of organisms and genome versions provided as support files.

| **Organism** | **miRNA** | **Genome version** | **Files** |
| --- | --- | --- | --- |
| Homo sapiens | hsa_mature.fa | hg19 | hg19.AGO.bed  hg19.ensGene.gtf  hg19.fa.gz  hg19.INT.bed  hg19.rRNA.bed |
|  |  | hg38 | hg38.AGO.bed  hg38.ensGene.gtf  hg38.fa.gz  hg38.INT.bed  hg38.rRNA.bed |
| Mus musculus | mmu_mature.fa | mm9 | mm9.AGO.bed  mm9.ensGene.gtf  mm9.fa.gz  mm9.INT.bed  mm9.rRNA.bed |
|  |  | mm10 | mm10.AGO.bed  mm10.ensGene.gtf  mm10.fa.gz  mm10.INT.bed  mm10.rRNA.bed |
| Drosophila melanogaster | dme_mature.fa | dm3 | dm3.AGO.bed  dm3.ensGene.gtf  dm3.fa.gz  dm3.INT.bed  dm3.rRNA.bed |
|  |  | dm6 | dm6.AGO.bed  dm6.ensGene.gtf  dm6.fa.gz  dm6.INT.bed  dm6.rRNA.bed |
| Caenorhabditis elegans | cel_mature.fa | ce10 | ce10.AGO.bed  ce10.ensGene.gtf  ce10.fa.gz  ce10.INT.bed  ce10.rRNA.bed |
|  |  | ce11 | ce11.AGO.bed  ce11.ensGene.gtf  ce11.fa.gz  ce11.INT.bed  ce11.rRNA.bed |
